# Artificial intelligence-assisted noninvasive preoperative prediction of lymph nodes metastasis in cervical cancer through a clinical-imaging feature combined strategy

**DOI:** 10.3389/fonc.2025.1669396

**Published:** 2025-12-17

**Authors:** Jingjing Zhang, Chunlong Fu, Junqiang Du

**Affiliations:** 1 Department of Gynecology and Obstetrics, Affiliated Dongyang Hospital of Wenzhou Medical University, Dongyang, Zhejiang, China; 2 Department of Radiology, Affiliated Dongyang Hospital of Wenzhou Medical University, Dongyang, Zhejiang, China

**Keywords:** cervical cancer, lymph node metastasis, artificial intelligence, multi-modal contrastive learning, predictive model

## Abstract

**Background:**

Lymph node metastasis (LNM) of patients with cervical cancer (CC) is correlated with noticeably reduced five-year survival rate. but the role of conventional detection is limited for preoperative diagnosis of LNM. Therefore, we intended to develop a predictive model for LNM by integrating medical images, clinical data along with artificial intelligence-assisted method.

**Methods:**

CC patients who underwent radical hysterectomy combined with pelvic lymphadenectomy between January 2013 and October 2024 were retrospectively enrolled in this study. For computed tomography (CT) and ultrasound (US) images, a pre-trained ResNet-18 model on large-scale samples was used to extract representative features, fine-tuned with random cropping data augmentation. For clinical indicators, after normalizing to the range [0,1], a multilayer perceptron block was applied to extract representative features. Then, contrastive learning and feature fusion methods were utilized to integrate similar messages. Finally, a multi-modal contrastive learning framework was developed by consolidating above two parts. The framework was estimated by accuracy, sensitivity, specificity and the area under the receiver operating characteristic curve (AUC).

**Results:**

This work consisted of 127 CT images of patients with pathologically diagnosed cervical malignancies. After integrating clinical-imaging feature and artificial intelligence-assisted algorithm, the finally developed LNM predicting model achieved a high accuracy of 92.31% with an AUC of 0.88. Additionally, the model also displayed strong sensitivity (80.0%) and specificity (95.45%) in CC cohorts.

**Conclusion:**

This study presented an efficient noninvasive and highly accurate diagnostic tool for LNM, which may significantly enhance surgical decision-making for lymph node dissection in CC patients with LNM.

## Introduction

Cervical cancer (CC), one of the four major malignant tumors affecting women, poses a significant threat to women’s health and lives worldwide (1). In 2020, there were approximately 604,000 cases and 342,000 deaths worldwide (2). Nonetheless, by 2022, the number has risen to 661,000 new cases and 348,000 deaths (3). Actually, lymph node metastasis (LNM) also has been identified as a dangerous factor that can strongly reduce the five-year survival rate of CC patients from 90% to 60.8% (4, 5). Therefore, the 2018 revision of the International Federation of Gynecology and Obstetrics (FIGO) staging system emphasized the significance of LNM in staging accuracy and clinical decision-making (6).

As a result, accurately assessing LNM is crucial for CC treatment. For instance, determining the precise status of stage IIIC1r patients at the initial treatment stage can prevent them from undergoing both surgery and chemoradiotherapy, thereby reducing unnecessary financial burden of treatment (7). Furthermore, for CC patients seeking fertility preservation, the 2023 National Comprehensive Cancer Network (NCCN) guidelines state that when patients with IA2-IB1 CC meet the ConCerv criteria could consider conservative surgery (*i.e.*, cervical conization with pelvic lymphadenectomy or sentinel lymph node mapping) (8).

Traditionally, pelvic lymphadenectomy has been a crucial step in examining LNM. However, the LNM rate in early-stage CC is below 20%, meaning that most patients do not benefit from routine pelvic lymphadenectomy and may face increased surgical risks, instead (9, 10). While the newly proposed sentinel lymph node mapping still lacks mountainous clinical reports (11). To this end, imaging technology plays a pivotal role in preoperative assessment and has been incorporated into the 2018 FIGO revision as a key metric in the staging system (6). However, in clinical practice, directly using imaging technology for LNM prediction presents several challenges: (i) the same imaging modality may demonstrate varying advantages depending on the specific task, making it suboptimal to rely on a single modality for LNM prediction; (ii) imaging technologies exhibit limited sensitivity in detecting micro-metastases, and their interpretation is subjective, potentially leading to missed or misdiagnosed cases; and (iii) some imaging modalities, such as MRI, are expensive and may not be widely accessible. For these reasons, and because the majority of patients in our retrospective cohort did not undergo MRI examinations, we focused on developing a model using the more universally available CT, ultrasound, and clinical data. This approach ensures broader applicability and avoids the significant sample size reduction that would result from requiring MRI for all patients.

As the progression of artificial intelligence (AI) technology in the field of computational pathology, AI-assisted multi-modal learning method is developed to help extract more extensive, substantial, and distinctive features through integrating images, tissue sections as well as texts (12). For example, Wei et al. (13) proposed a novel multi-modal learning framework for genotype prediction in Glioma. Magazine “Cell Reports Medicine” also newly published a paper concerning the method, which could accurately forecast stomach cancer response to neoadjuvant chemotherapy with noticeably reduced distances to tumor invasion margin and enhanced inflammatory infiltration in responders (14). Whereas, multi-modal learning method is rarely employed to LNM related research on CC patients.

Based on aforenoted backgrounds, we aim to purport a multi-modal contrastive learning framework for accurate LNM prediction in CC patients through integrating CT images, US images and laboratory indicators. This approach would present a novel direction for accurate LNM prediction in CC.

## Materials and methods

This was a single-center retrospective cohort design, enrolling consecutive CC patients who underwent radical hysterectomy combined with pelvic lymphadenectomy at Dongyang People’s Hospital, affiliated with Wenzhou Medical University (a tertiary teaching hospital in China), between January 1, 2013, and October 31, 2024. The study protocol was approved by the Institutional Ethics Review Committee (Approval No.: 2024-YX-362), with a waiver of informed consent due to its retrospective nature. All data processing strictly adheres to the principles of the Declaration of Helsinki, and original medical records are securely stored in the hospital’s electronic medical record archiving system.

### Patients enrollment

Patients were included based on the following criteria:

- Histopathological confirmation of CC, with diagnosis based on the 2018 FIGO staging system;
- Newly diagnosed cases, i.e., patients who have not received neoadjuvant chemotherapy or radiotherapy;
- Complete surgical records and pathological reports;
- Preoperative imaging assessment, including CT and US.

The exclusion criteria including:

- Concurrent diagnosis of other malignant tumors;
- Abnormal liver or kidney function tests (alanine aminotransferase (ALT)/aspartate aminotransferase (AST) > 2 times the upper normal limit, creatinine clearance < 60 mL/min);
- Presence of hematologic or immune system disorders, or acute/chronic infections;
- Missing key data.

**Figure 1:**
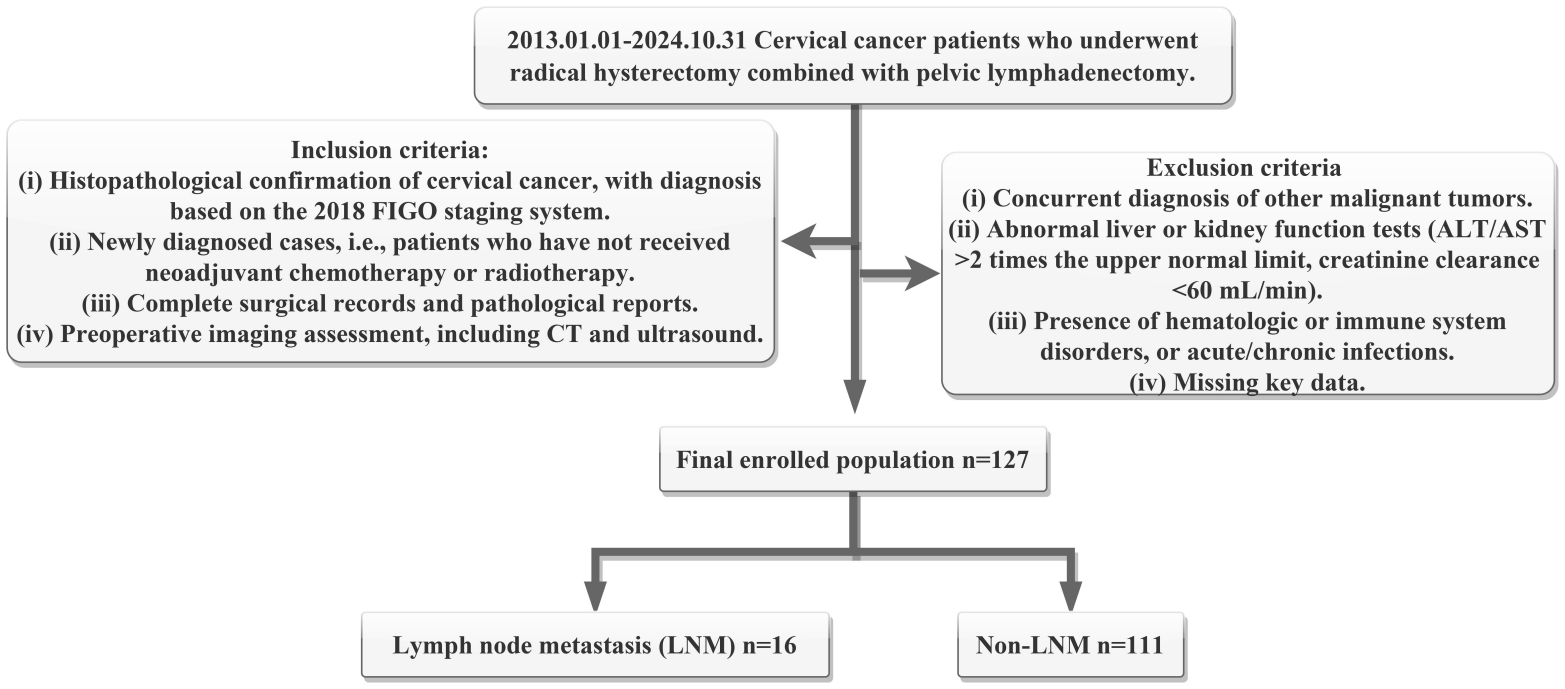
Flowchart of patient selecting criteria and enrolling number. Here, ALT means alanine aminotransferase, AST means aspartate aminotransferase, FIGO means the International Federation of Gynecology and Obstetrics, CT means computed tomography, LNM means lymph node metastasis.

After rigorous screening, a total of 127 patients were included in the final analysis (**Figure 1**). Among them, 16 cases with LNM were set as positive group, while 111 cases were classified as the negative group.

### Imaging acquisition

CT Scanning Protocol. CT Scanning was carried out in accordance to paper published before (15). Briefly, Patients were positioned in the supine position, with the scan range covering the region from the pubic symphysis to the iliac crest. Then, a 64-slice spiral CT scanner was used for both non-contrast and contrast-enhanced scans of the entire abdomen and pelvis with a slice thickness of 5 mm. An iopromide contrast agent (300 mg/mL, 1.5 mL/kg) was administered intravenously. The CT scanners used in this study included the Canon Aquilion One, Philips Brilliance, Siemens Force, and Siemens Somatom series, all of which offered high-resolution imaging and rapid scanning capabilities. All CT images were obtained from the institutional picture archiving and communication system and saved in DICOM format.

US Examination Protocol. Patients were placed in the lithotomy position, and transvaginal ultrasound was performed with a probe frequency of 4–8 MHz. The probe was carefully inserted into the vaginal fornix and gradually rotated to obtain multi-plane imaging. Key observations included cervical morphology, size, myometrial echogenicity, and endometrial characteristics. Additionally, a detailed evaluation of any detected masses was performed, focusing on external morphology, boundary definition, and internal echogenicity to comprehensively capture imaging features of cervical lesions. The US equipment used in this study included the PHILIPS EPIQ7W and Voluson E8, both of which provide advanced imaging capabilities for precise CC assessment.

### Laboratory data

Squamous Cell Carcinoma Antigen (SCC-Ag) Detection. SCC-Ag levels were measured using the MAGLUMI2000 chemiluminescence immunoassay analyzer, employing a sandwich immunoluminescence assay (ARCHITECT i2000SR, Abbott). The critical threshold for SCC-Ag was 1.8 ng/mL.

CA125 and CA19–9 Detection. CA125 and CA19–9 as common tumor markers (16, 17) were determined with the Cobas electrochemiluminescence immunoassay analyzer, applying a double-antibody sandwich electrochemiluminescence assay. The reference ranges were:

- CA125: < 35 U/mL;
- CA19-9: < 39 U/mL.

Complete Blood Count (CBC) Testing. CBC count was analyzed applying the XE-2100 automated hematology analyzer, including:

- Absolute neutrophil count: 1.8−6.3 × 109/L;
- Absolute lymphocyte count: 1.1−3.2 × 109/L;
- Platelet (PLT) count: 125−350 × 109/L.

Serum Albumin (Alb) Level. Serum Alb levels were tested by the Hitachi 7600–120 automated biochemical analyzer, based on the bromocresol green colorimetric method. The reference range was 40–55 g/L.

Inflammation/Nutritional Index Calculations. Based on the laboratory test results, the following indices were calculated:

- Neutrophil-to-Lymphocyte Ratio (NLR);
- Platelet-to-Lymphocyte Ratio (PLR);
- Systemic Immune-Inflammation Index (SII).

$$=\frac{\text{Platelet count}\left(\times 10^{9}/\text{L}\right)\times \text{Neutrophil count}\left(\times 10^{9}/\text{L}\right)}{\text{Lymphocyte count}\left(\times 10^{9}/\text{L}\right)\;}$$

• Prognostic Nutritional Index (PNI).


$$=\text{Albumin}\left(\text{g}/\text{L}\right)+\text{Lymphocytecount}\left(10^{9}/\text{L}\right)$$

Postoperative pathological diagnoses were independently reviewed and confirmed by two senior pathologists to ensure accuracy and reliability. The pathological reports included key parameters such as tumor size, histological type, differentiation grade, depth of invasion, LNM status (including the number and location of metastatic nodes), and surgical margin status.

### Model architectures

**Figure 2:**
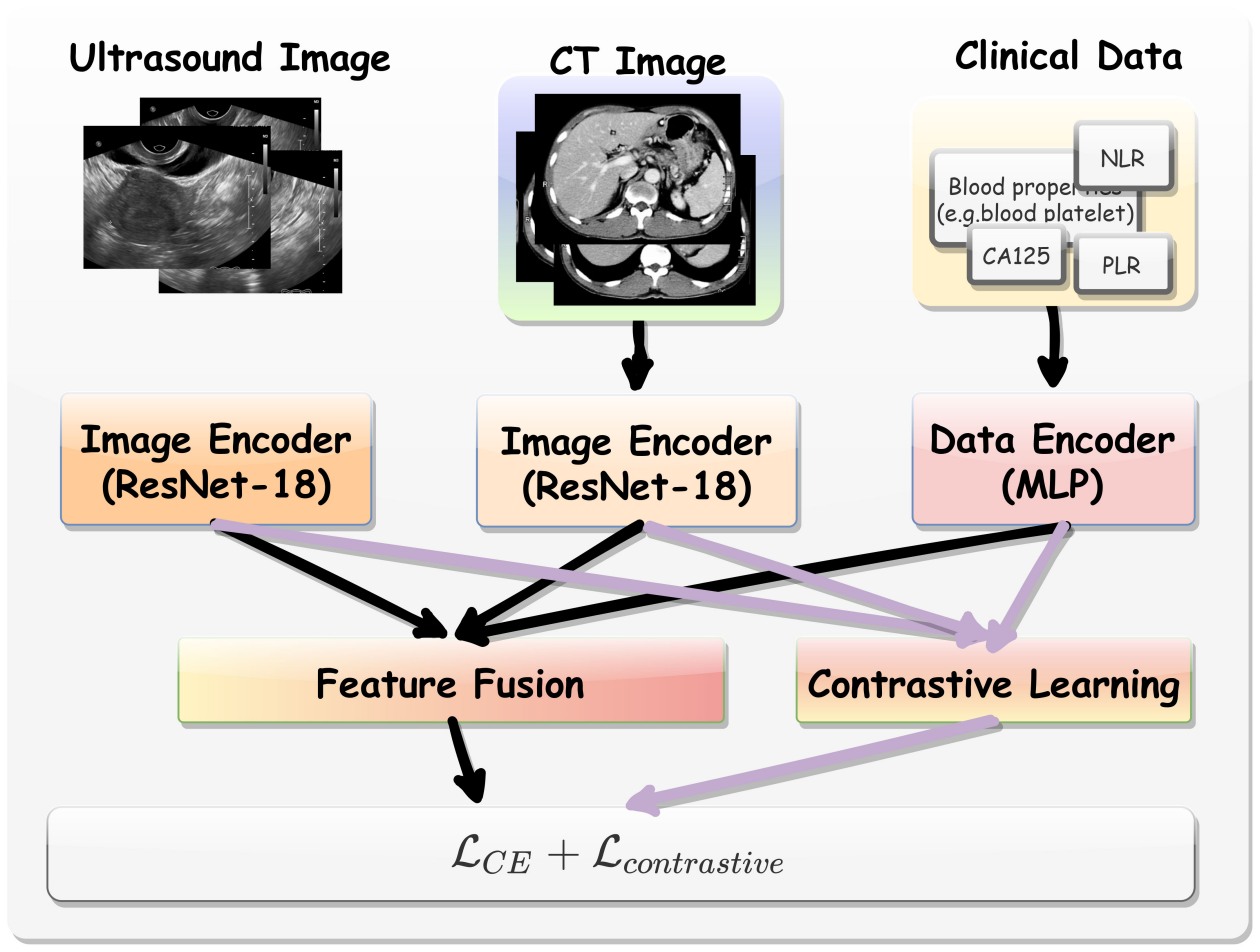
The overall analytical framework of multi-modal contrastive learning procedure.

The overall analytical framework was illustrated in **Figure 2**.

Multi-modal Data Encoding. Here, we employed a pre-trained ResNet-18 model (18) to extract features from CT and US images. This model was pretrained on large-scale publicly available datasets that include CT images of various target organs (19). As such, the backbone incorporates extensive medical knowledge and provides an effective starting point for fine-tuning on our target tasks, especially when training data for the limited downstream tasks. We extracted the hidden feature from ResNet-18 immediately before its final linear layer as the general CT and US image representation. Denoted by *H_ct_* ∈ ℝ*^D^* and *H_us_* ∈ ℝ*^D^* where *D* = 512. For clinical data, a multilayer perceptron (MLP) block comprising two linear layers was utilized. The first layer outputted a 256-dimensional feature vector, while the second layer projects the data outputted a 512-dimensional space (*H_clin_* ∈ ℝ*^D^*).

Contrastive Learning. The contrastive learning module was designed to align CT, US image features and clinical data features in a shared latent space. Given the extracted feature vectors from above two modalities, we optimized the model using an additional contrastive loss function (20) that encouraged positive pairs (*i.e.*, the same patient data) to be closer in feature space while pushing negative pairs (*i.e.*, different patients) apart. Specifically, for each patient *i*, we extracted CT and US image features as shown in Equation 1.

(1) $$H_{ct}^{\left(i\right)}=f_{ct}\left(X_{ct}^{\left(i\right)}\right)\in {\mathbb{R}}^{D};\;H_{us}^{\left(i\right)}=f_{us}\left(X_{us}^{\left(i\right)}\right)\in {\mathbb{R}}^{D}$$

$X_{ct}^{\left(i\right)}$ and $X_{us}^{\left(i\right)}$ were the raw CT and US image data from patient *i*, respectively. While, $H_{ct}^{\left(i\right)}\;$ and $H_{us}^{\left(i\right)}$ were feature vector extracted from $X_{ct}^{\left(i\right)}$ and $X_{us}^{\left(i\right)}$ with $\;f_{ct}$ and $f_{us}$ function from ResNet-18 model. In the same time, we also extracted clinical feature vectors were extracted using the multilayer perceptron block as described in Equation 2.

(2) $$H_{clin}^{\left(i\right)}=f_{clin}\left(X_{clin}^{\left(i\right)}\right)\in {\mathbb{R}}^{D}$$

To improve contrastive representation learning, we applied additional projection layers as defined in Equation 3.

(3) $$Z_{ct}^{\left(i\right)}={\text{g}}_{ct}\left(H_{ct}^{\left(i\right)}\right);\;Z_{us}^{\left(i\right)}={\text{g}}_{us}\left(H_{us}^{\left(i\right)}\right);\;Z_{clin}^{\left(i\right)}={\text{g}}_{clin}(H_{clin}^{\left(i\right)})$$

where *g*_CT_, *g*_us_ and *g*_clin_ were MLP-based projection heads that mapped the features to a lower-dimensional contrastive space, *i.e.*, 128 in our experiment. To maximize the similarity among a patient’s CT features, US features and clinical features while minimizing similarity between different patients’ feature pairs, we used the cosine similarity function defined in Equation 4.

(4) $$sim\left(Z_{a}^{\left(i\right)},Z_{b}^{\left(i\right)}\right)=\frac{Z_{a}^{\left(i\right)}\cdot Z_{b}^{\left(i\right)}}{\left\|Z_{a}^{\left(i\right)}\right\|\left\|Z_{b}^{\left(i\right)}\right\|},a\in \left\{ct,\;clin,\;us\right\},b\in \left\{ct,\;clin,\;us\right\},a\neq b$$

And the model was also optimized with the InfoNCE loss function to further maximize the similarity between positive pairs (21) as shown in Equation 5.

(5) $$ℒ\left(Z_{a}^{\left(i\right)},Z_{b}^{\left(i\right)}\right)=-\log\frac{\text{exp}(sim(Z_{a}^{\left(i\right)}\cdot Z_{b}^{\left(i\right)})/\tau )}{\sum_{j\neq i}\text{exp}(sim(Z_{a}^{\left(i\right)}\cdot Z_{b}^{\left(i\right)})/\tau )}$$

The overall contrastive loss is computed as described in Equation 6.

(6) $${ℒ}_{contrastive}=\frac{ℒ\left(Z_{ct}^{\left(i\right)},Z_{clin}^{\left(i\right)}\right)+ℒ\left(Z_{us}^{\left(i\right)},Z_{clin}^{\left(i\right)}\right)+ℒ\left(Z_{ct}^{\left(i\right)},Z_{us}^{\left(i\right)}\right)}{3}$$

where τ was a temperature scaling hyperparameter that controlled the sharpness of similarity scores, we use τ = 1 as the default in this experiment.

Optimization Objective. Apart from contrastive learning, the learned CT, US and clinical features were also fused together by concatenation as shown in Equation 7.

(7) $$H_{fusion}^{\left(i\right)}=concat(H_{ct}^{\left(i\right)},\;H_{us}^{\left(i\right)},\;H_{clin}^{i}\in {\mathbb{R}}^{3D})$$

This fused representation was then passed to a binary classifier with loss function for the final prediction. The overall training objective is shown in Equation 8.

(8) $$ℒ={ℒ}_{CE}\left(H_{fusion}^{\left(i\right)},Y_{label}\right)+{ℒ}_{contrastive}$$

Here ${ℒ}_{CE}$ means cross entropy loss, ${ℒ}_{contrastive}$ means contrastive loss. $Y_{label}$ equals 0 or 1, indicating the positive or negative property of patient’s LNM.

### Implementation details

To ensure a rigorous evaluation and mitigate overfitting, the entire dataset was randomly divided into three independent sets: training (80%), validation (10%), and testing (10%). The model parameters were optimized using the training set, and the model checkpoint that achieved the highest accuracy on the validation set was selected. All performance metrics reported in this study (e.g., in **Table 1**) are based solely on the held-out test set, which was not used at any stage of training or model selection.

**Table 1:** Clinicopathological characteristics of patients.

Characteristics	Total n percentile (n=127)		*P* value	Test statistic
	LN+ (n=16)	LN- (n=111)		
Age (years)	56.0 (49.5,63.8)	54.8 (48.0,64.0)	0.676	*z* = -0.418
SCC-Ag level			0.016	*x²* = 5.853
Normal (<1.8ng/ml)	5 (31.3%)	70 (63.1%)		
Elevated (≥1.8ng/ml)	11 (68.8%)	41 (36.9%)		
CA125 level			0.126	*x²* = 2.337
Normal (<35ng/ml)	13 (81.3%)	103 (92.8%)		
Elevated (≥35ng/ml)	3 (18.8%)	4 (3.6%)		
CA19–9 level			0.013	*x²* = 6.160
Normal (<39ng/ml)	13 (81.3%)	107 (96.4%)		
Elevated (≥39ng/ml)	3 (18.8%)	8 (7.2%)		
PLT (*10^9/l)	244.3 ± 48.3	245.0 ± 65.5	0.968	*t* = 0.040
Alb (g/l)	42.2 (40.1,46.9)	42.4 (40.3,44.9)	0.83	*z* = -0.214
NLR	3.2 (1.9,4.1)	2.9 (1.7,3.2)	0.383	*z* = -0.872
PLR	158.2 (129.6,173.8)	163.6 (113.6,184.1)	0.551	*z* = -0.596
SII	764.7 (430.4,1005.4)	693.3 (375.5,829.4)	0.372	*z* = -0.894
PNI	50.4 ± 5.1	51.0 ± 5.4	0.693	*t* = 0.396
Clinical stage			<0.001	*x²* = 92.045
FIGO I-II	3 (18.8%)	110 (99.1%)		
FIGO III-IV	13 (81.3%)	1 (0.9%)		
Differentiation Grade			0.909	*x²* = 0.013
Well/Moderately differentiated	10 (62.5%)	71 (64.0%)		
Poorly/undifferentiated	6 (37.5)	40 (64.0%)		
Pathological lymphovascular invasion status			0.061	*x²* = 3.520
Positive	8 (50.0%)	81 (73.0%)		
Negative	8 (50.0%)	30 (27.0%)		
Pathological perineural invasion status			0.559	*x²* = 0.341
Positive	14 (87.5%)	102 (91.9%)		
Negative	2 (12.5%)	9 (8.1%)		

To train our framework, The Adam optimizer with an initial learning rate of 1e-3, following a cosine annealing schedule for learning rate decay was carried out. The model was trained for 30 epochs, and all experiments were implemented using the PyTorch framework on NVIDIA A100 GPUs. To enhance data quality and improve generalization, Normalization and augmentation techniques were utilized. Firstly, both CT and US images underwent random cropping^1^ followed by resizing to a resolution of 224. Secondly, clinical data were converted into the range [0,1] to mitigate numerical instability and reduce biases induced by raw values. Lastly, we adopt mini-batch training with a batch size of 8, where the sampling probability was adjusted to ensure that underrepresented classes had a higher chance of being selected. This strategy was proved particularly effective for classification tasks with class imbalances.

### Validation and evaluation of models

To objectively evaluate the effectiveness of the approach in detecting LNM in CC cohorts, multiple performance indicators were adopted, containing accuracy $\left(\text{ACC}=\frac{TP+TN}{TP+TN+FP+FN}\right)$, sensitivity $\left(\text{SE}=\frac{TP}{TP+FN}\right)$, specificity $\left(\text{SP}=\frac{TN}{TN+FP}\right)$, and the area under the receiver operating characteristic curve (AUC). Here, TP, TN, FP, and FN were represented as true positives, true negatives, false positives, and false negatives, respectively.

### Statistical analysis

Clinical data were analyzed using SPSS version 26.0 (Statistical Package for the Social Sciences). The Kolmogorov–Smirnov (K-S) test was used to assess the normality of continuous variables. Normally distributed data were expressed as mean ± standard deviation (x ± s), and compared using the independent samples t-test. Non-normally distributed data were presented as median and interquartile range (P25, P75), with comparisons performed using the Mann–Whitney U test. Categorical variables were reported as frequency and percentage, and analyzed with the chi-square test or Fisher’s exact test, as appropriate.

## Results

### Patient characteristics

In this study, 127 eligible patients were divided into LNM-positive group (LNM+, n = 16) and LNM-negative group (LNM-, n = 111). The patient baseline characteristics and conventional diagnostic features were summarized in **Table 1**. No obvious differences were seen between LNM+ and LNM- patients in terms of age, CA125 level, PLT level, Alb level, NLR, PLR, SII, differentiation grade, pathological lymphovascular invasion status and pathological perineural invasion status between two groups (p > 0.05), indicating no easily apparent diagnosis for LNM. Only the SCC-Ag, CA19–9 and FIGO based clinical stage showed marked differences between the groups (p< 0.05, respectively).

### Predictive performance

The overall performance of this study was summarized in **Table 2**. The ablation study highlighted the impact of incorporating additional modalities and contrastive learning on prediction accuracy. Specifically, using a single modality, the highest accuracy achieved was 80.77%, with clinical data proving to be the most effective. This was likely because clinical data tended to be more precise, whereas CT and US images contained redundant information, requiring the model to filter out noise.

**Table 2:** Results of lymph node classification.

CT	US	Clinical	Contrastive	ACC (%)	SE (%)	SP (%)	ROC (%)
✓	✗	✗	✗	76.92	60.00	80.95	71.42
✗	✓	✗	✗	73.08	60.00	76.19	79.04
✗	✗	✓	✗	80.77	60.00	85.71	84.96
✓	✓	✓	✗	84.62	60.00	90.48	86.67
✓	✓	✓	✓	92.31	80.00	95.45	88.17

A checkmark indicated the use of this type of data, while a cross implied that this type of data is not applied. CT, using CT data; US, with ultrasound US data; Clinical, with applying Clinical data; Contrastive, employing contrastive learning; ACC, accuracy; SE, sensitivity; SP, specificity; ROC, receiver operating characteristic.

**Figure 3:**
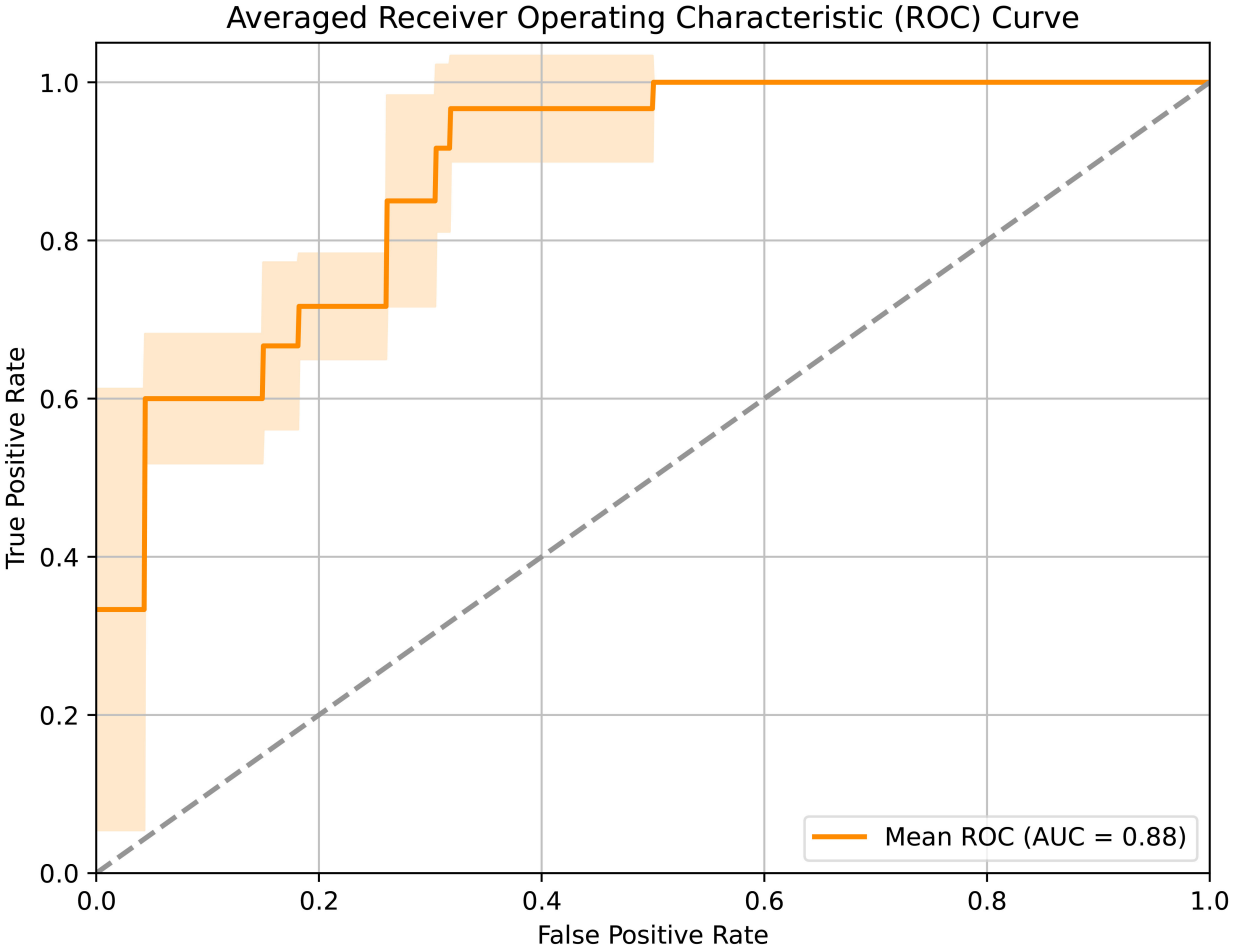
Receiver operating characteristic curve of the developed framework.

When integrating all above three modalities, the accuracy was improved to 84.62%. Noticeably, by leveraging multi-modal data and contrastive learning method, our model achieved an impressive 92.31% accuracy in LNM prediction, demonstrating its strong efficacy. The corresponding AUC curves in **Figure 3** further validated the effectiveness of our approach, with a high AUC of 0.88.

Notably, the ablation study (upper part of **Table 1**) revealed that clinical data alone provided the highest predictive performance among single-modality inputs. This observation aligns with clinical intuition, given that laboratory markers such as CA125, NLR, and PLR are established correlates of lymph node metastasis, thereby offering interpretability into the model’s decision process.

## Discussion

This study novelly established a predicting model based on multi-modal contrastive learning to assess the occurrence of LNM in CC patients. The model addressed the limitations of traditional methods and offered an efficient, non-invasive tool for preoperative evaluation, thereby supporting clinical decision-making and improving patient outcomes.

Current LNM evaluations rely primarily on imaging modalities. However, these methods face two major challenges: (1) image interpretation depends heavily on physician expertise, inducing subjectivity; and (2) limited sensitivity in detecting micrometastases (*e.g.*, <5 mm) (22). To address these challenges, the integration of artificial intelligence and medical imaging offers a promising solution. While traditional machine learning methods show clinical potential, their reliance on manual feature engineering (e.g., regions of interest annotation) limited its practical utilization (23). Recently, advancements in unsupervised learning, deep feature extraction and multi-modal feature fusion have demonstrated promising improvements of enhancing model capability (24–26).

Motivated by recent advances, this study integrated CT and US imaging with a range of clinical data—including tumor markers (CA125, SCC, CA19-9) and emerged prognostic indices such as SII and PNI based on a systemic review (27). CT and US were chosen as the primary imaging modalities due to their cost-effectiveness and diagnostic performance, which is comparable to MRI for evaluating LNM and parametrial invasion (24). Through multi-modal integration, the model captures complementary information across data sources, boosting prediction accuracy from 76.92%-80.77% in single modality to 84.62% in three combined modalities, accompanied with increased ROC from 0.71-0.84 to 0.86. Furthermore, given the highly imbalanced nature of our dataset, there was a risk that the model could be biased toward predicting the majority class. However, by employing a weighted sampling strategy during training, our model achieved high sensitivity and specificity, indicating that the model effectively distinguished true labels rather than defaulting to majority-class predictions. Machine learning models generally require large-scale, high-quality data for accurate prediction. So, one of the primary challenges for accurately prognosticate LNM in CC is the limited available training data. To address this limitation, a pre-trained model (19) on a large-scale public medical dataset was adopted to acquire general medical representations. Additionally, to make effective predictions, we also proposed several strategies to fully leverage the available data, including the integration of multi-modal data and the application of contrastive learning.

Moreover, this study employed a contrastive learning framework that mapped heterogeneous modalities into a shared latent space. By maximizing similarity between positive pairs and minimizing it between negative pairs using the InfoNCE loss function, the model extracted discriminative cross-modal features. This approach significantly enhances performance, raising accuracy to 92.31% and AUC to 0.88. This result was better than previously published LNM prediction model in pancreatic cancer (74.4%, 0.79) (28) as well as newly published similar paper in CC (87%, 0.81) (29). To enable a direct comparison on our dataset, we reproduced a closely related baseline model for cervical cancer LNM prediction (29). Our proposed framework attained a higher accuracy of 92.31% compared to 87.25% achieved by this baseline, highlighting the effectiveness of our multimodal contrastive learning approach in integrating complementary information. This improvement highlights the effectiveness of our method in integrating complementary information from CT, ultrasound, and clinical data to enhance lymph node metastasis prediction performance.

The proposed multi-modal contrastive learning model holds substantial clinical value. First, accurate preoperative LNM prediction can help avoid unnecessary surgical interventions, reducing treatment burden. Beyond the acute complications, the choice of treatment modality has profound implications for long-term quality of life. As highlighted by Di Donna et al., multimodal treatments for cervical cancer, including radical surgery and chemoradiotherapy, are frequently associated with long-term urinary, gastrointestinal, and sexual dysfunctions (30). Our non-invasive predictive model aids in refining clinical decision-making. Second, including US as a data source provides a more accessible and cost-effective solution, particularly beneficial in low-resource settings where US is more available than other imaging equipment. Moreover, the model’s high sensitivity and specificity enhance its reliability in handling imbalanced datasets, which is common in real-world applications.

Despite the promising results, this study has several limitations. It was a single-center retrospective study with a limited sample size, which may affect the generalizability of the proposed method. To partially address this, we employed a pretrained ResNet-18 model trained on large-scale medical datasets to enhance generalization capability. Furthermore, to evaluate robustness under single-center data constraints, we conducted supplementary experiments assessing cross-age generalization and resilience to Gaussian blur noise that simulated multi-center imaging variability. The model maintained strong performance (e.g., accuracy >85%, AUC >81%) across these challenging conditions, underscoring its robustness.

Nonetheless, this preliminary effort yielded encouraging outcomes. Future work will include multicenter validation with larger cohorts. Additionally, although contrastive learning contributed to performance improvement, external validation is essential to further assess its clinical utility. Finally, this study focused exclusively on CT, US, and clinical data. Subsequent research will aim to incorporate additional imaging modalities such as MRI and PET to further enhance performance.

## Conclusion

In conclusion, this study built an efficient LNM prediction strategy in CC patients using a multi-modal contrastive learning approach along with a pre-trained model on large-scale samples. The model demonstrated excellent predictive performance and hold significant potential for clinical application. It is expected to provide more accurate diagnostic and therapeutic guidance for CC patients, optimize treatment decisions, and improve patient outcomes.

## Data Availability

The raw data supporting the conclusions of this article will be made available by the authors, without undue reservation.

## Footnotes

1 https://pytorch.org/vision/main/generated/torchvision.transforms.RandomCrop.html.
